# Effectiveness of plants and green infrastructure utilization in ambient particulate matter removal

**DOI:** 10.1186/s12302-021-00547-2

**Published:** 2021-09-25

**Authors:** Katarzyna Wróblewska, Byoung Ryong Jeong

**Affiliations:** 1 Department of Horticulture, Wrocław University of Environmental and Life Sciences, Wrocław, Poland; 2 Department of Horticulture, College of Agriculture and Life Science, Gyeongsang National University, Jinju, 52828 South Korea; 3 Division of Applied Life Science (BK21 Four), Graduate School, Gyeongsang National University, Jinju, 52828 South Korea; 4 Institute of Agriculture and Life Science, Gyeongsang National University, Jinju, South Korea

**Keywords:** PM capture, Vegetation, Green roofs, Living walls, Urban farming

## Abstract

Air pollution is regarded as an increasingly threatening, major environmental risk for human health. Seven million deaths are attributed to air pollution each year, 91% of which is due to particulate matter. Vegetation is a xenobiotic means of removing particulate matter. This review presents the mechanisms of PM capture by plants and factors that influence PM reduction in the atmosphere. Vegetation is ubiquitously approved as a PM removal solution in cities, taking various forms of green infrastructure. This review also refers to the effectiveness of plant exploitation in GI: trees, grasslands, green roofs, living walls, water reservoirs, and urban farming. Finally, methods of increasing the PM removal by plants, such as species selection, biodiversity increase, PAH-degrading phyllospheric endophytes, transgenic plants and microorganisms, are presented.

## Background

Human activity plays a central role distort many of Earth’s regular functions. Intensification of these changes and their impact on nature and humans has led to Paul Crutzen to name the present epoch “Anthropocene” [1]. Its beginning is usually related to the Industrial Revolution in the eighteenth and nineteenth centuries when energy-consuming processes, like mechanization or reactive nitrogen syntheses, have started [2], which brought about intensive fossil fuel mining and coal combustion, accompanied by increasing population and urbanization. This resulted in emissions of black smoke, i.e., a composition of carbonaceous particles as major components of the airborne particulate matter (PM), carbon dioxide (CO_2_), sulfur dioxide (SO_2_), nitrogen oxides (NO_x_), and water vapor. PM react with water to form new particles with different properties and lead to periodic smog, which has been well-documented in London in 1952, which led to increased mortality from pneumonia and bronchitis [3]. Alongside the development of agriculture, increased methane (CH_4_) contents has been observed [4]. Moreover, CO_2_, CH_4,_ N_2_O, and vapor present in the air, have been observed to be responsible for the depletion of the stratospheric ozone layer, which results in the greenhouse effect and global warming, which is one of the greatest ecological concerns of the contemporary world.

Further rapid increases in air pollution and greenhouse effects have been noted since the 1950s, brought about by the explosive population growth, technological development, and prolonged life expectancy. The Great Acceleration, as it is called, is also a period of unprecedented rise of consumption, overusing natural resources and over-producing harmful byproducts. With the switch from coal to oil combustion, the concentration of black smoke has dropped [3], but new contaminants have been added to the atmosphere. Their presence is connected with industrial combustion, automobile transport, as well as the accelerated development of the chemical and pharmaceutical industries [5]. Other sources of air pollution include power plants, waste (especially combusted waste), as well as household heating, cooking and lighting [6]. They ‘enrich’ the air of metals, metalloids, hydrocarbons (HCs), including chlorofluorocarbons (CFCs), hydrofluorocarbons (HFCs), halons containing Br, volatile organic compounds (VOCs), and others. Cl, F and Br ions released from HCs and halons catalyze thousands of reactions with O_3_ in the stratosphere, accelerating the greenhouse effect [7].

All the previously mentioned contaminants form mixtures that pose danger to human health. In fact, air pollution is regarded as the main environmental risk for human health and is a rising concern [8]. After tobacco smoking, air pollution is the second most common form of noncommunicable diseases. Globally, air pollution accounts for 7 million premature deaths every year, 91% of which is caused by particulate matter [9]. The most ubiquitous death causes include stroke, heart disease, genotoxic effects such as leukaemia, lymphomas, lung cancer, and respiratory diseases such as asthma. Higher morbidity embraces also other cardiovascular and respiratory problems, but also kidney, intestinal, nervous (including depression, dementia and Alzheimer disease) disorders, allergy, and inflammation through the generation of reactive oxygen species and oxidative stress [10, 11]. All of them lead either to deterioration of well-being, or vast economic costs. In fact, economic costs are far beyond the medical problems related to pollution. Among others, dust, impaired visibility, air traffic distortions, odor, decreased agricultural yield, and degraded quality of products (e.g. incomplete ripening of fruits) due to reduced solar irradiation are mentioned as economic costs related to air pollution. Indirect costs connected with climate change, like extreme heat incidents, increased UV radiation, decreased or immense precipitation, water availability, floods and desertification bringing about further health problems, damage of property and infrastructure, and reduction of agricultural production are also associated with air pollution. Another concern is an accelerated sea level rise which threatens low-lying islands and coastal zones [7, 12, 13]. The ecological costs related to air pollution is difficult to evaluate.

According to the WHO [14], 91% of the world’s population breathe air that’s polluted beyond safe levels, which is 10 μg m^−3^ annually and 25 μg m^−3^ daily for PM_2.5_, and 20 μg m^−3^ annually and 50 μg m^−3^ daily for PM_10_ [14]. The very fatal phenomenon of air pollution is its relationship with the economic development of a country following the Kuznetz curve [15, 16], which means that countries with lower income, which are often overpopulated and suffering from inadequate health care system, experience a higher level of air contaminants. Among numerous ways of air pollution mitigation, including political policies and technological methods focusing on reduction of emissions (by reduction of energy consumption and increasing urban sustainability) [17], programs employing massive tree and shrub planting are recommended [14, 18] as they are considered cheap, eco-friendly and multi-beneficial [19]. It also brings about positive side effects connected with climate change mitigation. Cities based on garden-city concepts such as Putrajaya, Malaysia, or cities with an intensively developing green infrastructure like Stuttgart, Germany, succeed in the attenuation of urban heat islands [20]. Studies from Asian cities like Kuala Lumpur, Singapore, and Hong Kong demonstrate that vegetation may reduce air temperature by as much as 4 °C [21].

**Fig. 1:**
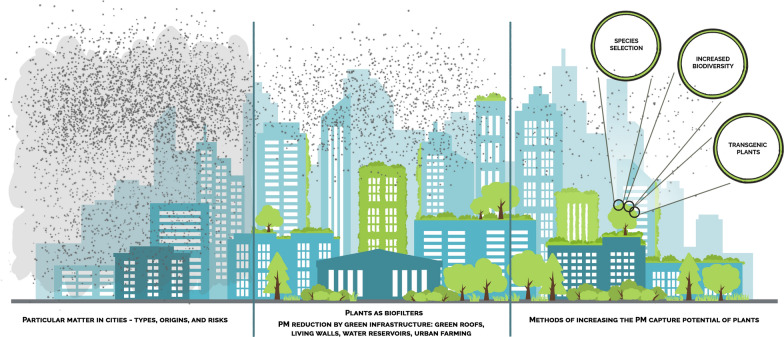
The structure of the article

According to Crippa et al. [22], in 2012 people emitted about 103 Mt of SO_2_, 562 Mt of CO, 122 Mt of NO_x_, 170 Mt of NMVOC, 59 Mt of NH_3_, 65 Mt of PM_10_ and 41 Mt of PM_2.5_ globally. How much of these residues can be removed and retained by vegetation? Is it possible to increase this amount and substantially improve the air quality? The purpose of this paper is to present a critical review on the possibilities of how plant utilization can effectively clean the air of the most hazardous contaminants, PM. In this paper we try to estimate the potential of plants to internally accumulate pollutants. We also consider factors affecting their effectiveness in order to find the conditions that should be fulfilled to improve the effectiveness of vegetation in air bioremediation (Fig. 1).

## Discussion

### The types of air pollutants, their origins, and risks

#### Physical stages of air contaminants: gases and aerosols

##### Gaseous pollutants

Within the main gaseous pollutants CO, NO_x_, SO_2_, total volatile organic compounds (VOCs), and NH_3_ are mentioned. Most gaseous pollutants are produced by human activity: road transport (NO_x_), fuel combustion (CO, SO_2_ and VOCs), industry (SO_2_), agriculture (NH_3_), off-road transportation, and the residential activities. SO_2_ and NO_x_ react with water in the atmosphere and generate acid precipitations. This phenomenon leads to deterioration of human health, degradation of plants, acidification of freshwater, deaths of aquatic animals, and damage of properties (corrosion of metals and weathering of stone buildings and monuments). This was the impetus for numerous policies aiming to lower the SO_2_ and NO_x_ emissions and gradually reduce global SO_2_ emissions since the 1980s. Contrary to this, global NO_x_ emissions are growing, although the trend varies among countries. The growing emissions in Asia, South America, Middle East and Africa contrast with the trend in Europe, where emissions are decreasing [23].

*Volatile organic compounds (VOCs)* are a group of miscellaneous compounds with a high vapor pressure due to a low boiling point within the range of 50–260 °C. Most VOCs are volatile at room temperature. VOCs include acetone, aliphatic hydrocarbons, aromatic hydrocarbons (benzene, toluene, ethylbenzene, xylenes and 1,2,4-trimethylbenzene), polycyclic aromatic hydrocarbons (PAHs), chlorinated compounds, and terpenes. They are a source of various scents and odors, and irritate the human skin and/or the respiratory system. They can cause cancer (i.e. benzene, ethylbenzene, tetrachloromethane, and trichloroethylene), allergy, and immune system impairments [24]. They are ubiquitous as they originate from different chemical products of everyday life: construction materials, paints, solvents, cleaning products, cosmetics, and others. The environmental risks posed by VOCs are based on their photochemical reactivity with anthropogenic NO_x_, which leads to the formation of ozone, secondary organic aerosols (SOA) and atmospheric oxidants (e.g. peroxyacil nitrates, aldehydes, ketones, hydrogen peroxide) [25]. Apart from anthropogenic sources (AVOCs), VOCs are also generated from plants and other organisms (BVOCs). Annual global VOC emissions are estimated to total about 100 Tg C, out of which the share of BVOCs is calculated to be about 90% [26].

##### Aerosols

Aerosols consist of liquid and solid particles (particular matter, PM) suspended in the air, and has a diameter between 0.001 and 100 µm. They are regarded as an equivalent to the historic black smoke, which now also refers to one of the methods of its measurement, i.e. its optical absorption (‘blackness’) [27]. PM molecules have different origins and diverse chemical properties. It consists mainly of black carbon dust, water-soluble ions (sulfates, nitrates, ammonia, chlorides), metals and organic pollutants, where some compounds are highly toxic to living organisms. Apart from chemical character, their size and surface area are key factors determining their malice. Usually, two categories of PM are monitored according to their aerodynamic diameter: PM_10_ and PM_2.5_. Their size determines the depth of deposition in the respiratory system [28]. *Coarse particles* (PM_10_,), with a diameter of 10 µm or less reach mainly the upper part of the respiratory system and can be removed by natural defense mechanisms. *Fine particles* (PM_2.5_), with the a diameter smaller than 2.5 µm, are inhaled into the lungs. PM_2.5_ is more hazardous to humans because of their greater surface area relative to their volume [29]. What is more, toxic polycyclic aromatic hydrocarbons (PAHs) are found within PM_2.5_, including the highly carcinogenic benzo(a)pyrene [30]. Within PM_2.5_, *ultrafine particles*, with aerodynamic diameters below 0.1 µm are also distinguished. These particles can cross the border of pulmonary vesicles and get to the blood. As they can behave like gases and enter organisms via diffusion, they can be absorbed also through ingestion, or through the skin, thus interfering with the functioning of different tissues and organs [31, 32]. Much of PM_2.5_ originates from fuel combustion and traffic. Its concentration in the air is less locally diversified as it can be suspended in the air for a longer time and transported with wind over long distances [33]. They contain several groups of compounds:

*Carbonaceous materials* Due to their origin of ubiquitous combustion sources, carbon particles are the most numerous in urban PM. The mass of carbonaceous material (total C, TC) account for up to 50–70% of PM_0.1_ [34]. Carbonaceous materials can be classified into two classes: black or elemental carbon (BC, EC) with a graphite structure, which is emitted directly from combustion, and organic carbon (OC), which contain different organic compounds, such as hydrocarbons.

*Persistent organic pollutants (POPs)* contain toxic chemicals which accumulate in organisms and the environment. As their half-life can be decades, they remain in the environment for a long time and can be transported over long distances; they have thus become a global concern and the motivation for the Stockholm Convention in 2001. According to the Convention, production of 12 chemicals, named the ‘DirtyDozen’—aldrin, chlordane, DDT, dieldrin, dioxins, endrin, furans, heptachlor, hexachlorobenzene, PCBs, and toxaphene—are especially hazardous to life and the environment, and their emissions were regulated [35]. 16 other chemicals were added to the list in 2017. POPs can be intentionally (e.g. in chemical industry) or unintentionally produced as byproducts of fuel combustion, waste, biomass, and industry. Among the most common airborne POPs, polycyclic aromatic hydrocarbons (PAHs), polychlorinated biphenyls (PCBs), polychlorinated dibenzo-p-dioxins and dibenzofurans (PCDDs/PCDFs), dioxin-like PCBs (dl-PCBs), as well as dichlorodiphenyl trichloroethane and its transformation products (DDTs) are mentioned [36].

Polycyclic aromatic hydrocarbons (PAHs) belong to the most ubiquitous air pollutants. PAHs are a group of hydrocarbons consisting of two or more fused aromatic rings. They can be divided into two classes: low-molecular-weight PAHs with 2 or 3 rings with a volatile character, and high-molecular-weight PAHs which contain 4 or more rings, which are significant constituents of PM_2.5_. As products of incomplete combustion of hydrocarbons, they are ubiquitous in the environment: they come from transportation, as well as industrial, domestic, agricultural and natural sources. The extraordinary risk of PAHs relates to their high carcinogenicity and toxicity, as well as their persistence due to their hydrophobicity, stability, and high sorption capacity. They are also resistant to biodegradation. The most ubiquitous in air, and the most hazardous is benzo(a)pyrene (BaP). It is usually used as a marker, and the risk coming from other PAHs is evaluated as benzo(a)pyrene equivalent (B(a)Peq) [37].

*Heavy metals* The presence of metals in the air is one of the major current concerns, both for environmental and health reasons. Most metals and metalloids constitute PM_2.5_, and those that do not have a diameter smaller than 0.1 µm. As some of them originate from rock erosion or volcanic eruption, they are ubiquitous in the Earth’s crust. Yet, metals from anthropogenic sources, including industrial (metallurgic, electroplating and tannery) and agricultural activities as well as vehicle transportation total to more than 5 times the amount of those released from rocks and soil. Some metals (Cu, Ni, Zn, Co, Fe and Mo), being a component of enzymes and pigments, are crucial for plant metabolism, but all metals are toxic in high concentrations. They may replace metals in pigments, disturb enzyme activities, and produce reactive oxygen species [38]. Cd, Pb and Hg seem to be the most hazardous for human health. They can have toxic, carcinogenetic, teratogenetic, neorotoxic and mutagenetic effects [39].

#### Origin of air pollutants

Contaminants get to the atmosphere in different ways. *Primary pollutants* are emitted directly to the atmosphere, whereas *secondary pollutants* develop in the air as a result of chemical or photochemical reactions [40]. Primary pollutants include gases like CO, NO_x_ and VOCs, as well as aerosols, such as black carbon or PAHs. Among the latter, ozone is the most ubiquitous pollutant. Both types may originate not only from human activities, but also from nature.

##### Anthropogenic sources

Anthropogenic sources contain mostly emissions from fuel combustion, traffic components, industry, and agricultural production. Burning solid fuels such as coal, wood, dung, and other biomass releases NO_x_, SO_2_, CO_2_, CO, PAHs, VOCs, and many others depending on the material they originate from. Coal is the most common energy source and is also the most hazardous, especially in residential areas in developing countries. The simple stoves used in those areas cause incomplete combustion, and discharge increased amounts of fine and ultrafine particles: Cu, Fe, S, carcinogenic arsenic, silica, fluorine, and metals, e.g. Pb, Ni, Cr, Hg, as well as highly toxic dioxins and dibenzofurans, acrylonitrile, benzo(a)pyrene, methyl bromide, and many others [28]. Combustion is a common method of agricultural land management. It includes burning of living and dead biomass, and deforestation for plant and animal production. The latter, apart from other ecological effects like biodiversity loss, defaunation, and global warming, releases to the atmosphere vast amounts of carbon that’s been stored over hundreds of years [41], as well as sulfates, nitrates, ammonia, and sodium chloride. For example, the expansion of agriculture into the tropical forest, commonly in the form of cattle breeding and oil palm plantations, was responsible for more than 2.6 Gt of CO_2_ year^−1^ (data from 2010–2014) [42]. After fossil fuel burning, deforestation is considered to be the second biggest source of anthropogenic greenhouse gases (GHGs) [43]_._ Other ubiquitous source of diverse air pollutants is transport, employing oil charged engines (vehicles, ships, railway and airplanes) and industry. Two further sources of PM pollutants, such as military conflicts and fireworks are also of great important and we should make a great effort to avoid them.

##### Natural sources

Natural sources of pollutants comprise of biological and geological sources, like volcanic activity, dust movement (e.g. sand storms), oceanic release, and wildfires. Biological contaminants may also include ubiquitous allergens, like pollen grains, spores, plant fibers, as well as parts of animal hairs and skin. Hairs of some plants, like *Heracleum sosnowskyi* Manden. suspended in the air may be strongly irritating and lead to photoallergy. Live and dead bacteria, fungi, and viruses are also present. What is more, bacterial endotoxins and mycotoxins may be involved in diverse inflammations. Gaseous pollutants come from lightning, soil emissions, mineralization of organic materials, fresh and marine water, forest fires, and others. The natural gases of major concern include biogenic volatile organic compounds (BVOCs) in prevalent amounts generated by plants, leading to the formation of ozone, SOA, and others [25]. BVOCs play a crucial role in signaling between plants and the environment (for example, as attractants for pollinators and distractions for herbivores) and may include, for example, isoprene, ethylene, fatty acids, and constituents of essential oils. They can be emitted as integral components in plant metabolism, or as a response to stresses [44, 45]. Constitutive compounds consist of, in majority, isoprene and monoterpenoids. Similarly, stress-induced BVOCs (sBVOCs) include monoterpenes, sesquiterpenes, sesquiterpenoids, benzenoids, and volatile lipoxygenase products (green leaf volatile products). BVOC emissions are positively correlated with the leaf area and stresses, like herbivore attacks, temperature, drought, and ozone content increase [46]. In cities, plants face many stresses like urban heat island (UHI), low humidity, drought, modified soils and pure aeration, as well as air and soil pollution. In such conditions, BVOC emissions rise substantially, up to about 100–1000 times that in non-stressed laboratory conditions [45]. These findings are corroborated by analyses of global BVOC emissions. European studies indicate that BVOC production is much higher in the Mediterranean region than in the northern countries [47]. Together with global warming, a further increase in the stress-induced BVOC production is expected (Sicard et al. 2016). Furthermore, sBVOCs, especially sesquiterpenes and benzenoids, have a greater potential for SOA formation than isoprene and monoterpenes do [45]. Global emissions of BVOCs is estimated to be 1000 Tg in (data from 2000), 50% of which belongs to isoprene and 30% to methanol, ethanol, acetaldehyde, acetone, *α*-pinene, *β*-pinene, *t*-βocimene, limonene, ethylene, and propene [48]. This composition varies around the world depending on the climate and plant species. For example, tropical rainforests, which cover about 18% of the globe’s area, are responsible for 80% of the global terpenoid emissions and 50% of other BVOC emissions [48].

### Plants as biofilters

#### Mechanisms of pollutant capture and detoxification

Plant metabolism is based on the gas exchange between plant tissues and the environment. It takes place through the stomata for most species, which exist on the abaxial side of the leaves. The aperture of the stomata reaches 4–10 nm and depends on several factors, including water availability, temperature, the stage of plant development, and the time of the day. The same factors influence absorption of gas and liquid xenobiotics as stomata are the main route through which they enter the leaf. Ultra-fine PM may also get to the plant through these doors. For lipophilic pollutants, like aromatic hydrocarbons, the cuticle is another pathway [49, 50]. After entering the leaf, the xenobiotics can be submitted to different processes to protect the organism against contaminant toxicity: excretion, conjugation, followed by compartmentalization or degradation to simple cell metabolites. Conjugation is a predominant and ubiquitous pathway. During this process contaminants are linked to cell compounds such as proteins, saccharides, lignin, amino acids, organic acids, and others [49], forming covalent bonds. This substantially decreases the toxicity of pollutants and allows them to be left in cells for a certain period until the plant enzymatic system is able to further transform them. The other possibility is compartmentalization, where cells remove the conjugates far away from strategic organelles, like nuclei, mitochondria and plastids for long-time storage. This process is thus also called ‘storage excretion’. Water-soluble conjugates are stored in vacuoles, while lipophilic ones accumulate in cell walls. Some of them can also be excreted to the extracellular spaces [51].

Contrary to gaseous pollutants, PM is mainly deposited on the leaf surface via wet or dry deposition. Adaxial surface of leaves collects more PM than the abaxial side does. For example, the average particle leaf surface density for adaxial side of *Platanus acerifolia* (Aiton) Willd. was twice as high as that for the abaxial side (3.4 × 10^4^ particles × mm^−2^ to 1.7 × 10^4^ particles × mm^−2^, respectively) [52]. In another study, 10–50% of the adaxial leaf surface area of *Ulmus pumila* L., *Salix babylonica* L. and *Ginkgo biloba* L. was covered by PM, compared to only 3–35% of the abaxial area [53]. This also impedes PM access to leaves via the stomata. Instead, the PM may be collected on the leaf surfaces or encapsulated in the cuticle. Nevertheless, contaminant accumulation on leaves is only a temporary capture, which ends with the falling of leaves. Then the contaminants transform into soil residues. Due to the longer period of storage (up to 5 years), evergreen species are generally more advantageous in air pollution mitigation [54]. Dry deposited particles can be removed from the surface by wind and rain. This is less likely if contaminants are embedded in the cuticle. What is more, some of lipophilic particles immobilized in the cuticle may be transported further into the parenchyma cells, where they are detoxified. It has been observed that hydrophilic contaminants, containing polar compounds and ions, can also penetrate the cuticle, but at far less intensive rates [55].

#### Factors affecting the efficiency of plant PM capture

Deposition of air pollutants in plants depends on diverse factors calculated according to the equation below, indicating the factors that influence this process [56]:

$$ {\text{Deposited}}\;{\text{amount}}\left( {{\text{g}} \times {\text{m}}^{{ - 2}} } \right) = {\text{LAI}} \times v_{{\text{d}}}  \times C \times t, $$

where LAI refers to the leaf area index; *v*_d_ is the deposition velocity, *C* is the concentration of the pollutant, and *t* is the time. Deposition velocity is usually calculated as the reciprocal of resistance to deposition, including aerodynamic, boundary and, the most impactful, surface resistance:

$$ v_{{\text{d}}}  = \frac{1}{{R_{{{\text{tot}}}} }} = \frac{1}{{R_{{\text{a}}} }} + \frac{1}{{R_{{\text{b}}} }} + \frac{1}{{R_{{\text{c}}} }} $$

This indicates that factors related to plant interaction with atmospheric pollutants are as follows: aerodynamic resistance, *R*_a_ depends on meteorology, whereas boundary (*R*_b_) and surface resistance (*R*_c_) depend on the friction and shear of the leaf surface as well as the size of the pollutant particle [56].

##### Factors connected with plants

Plant foliage acts as a sink for PM deposition. At least in part, it is attributed to electromagnetic charge of the leaf surface, connected with the plant taxa. A species’ ability to immobilize air contaminants is regarded as the key factor connected with plants themselves. Among the most relevant traits of a plant species’ ability to capture airborne pollutants are the leaf area index (LAI), morphology, anatomy (the complexity of the leaf surface and structure, the thickness of the wax layer, the presence of trichomes, etc.), and the stomatal density [53, 57–59]. These features are involved with the wettability and resistance of the leaf against being “occupied” by the pollutant particles. They are also responsible for the strength with which pollutant particles are attached to the leaf and not re-suspended in the air or washed by precipitation.

*Leaf area index (LAI)* LAI expresses the ratio of the leaf area to the area of the ground surface. Trees, with the largest LAI, are the most effective aerosol depositors [58, 60]. More expressed LAI can be found among trees with small (e.g. *Betula pendula* Roth) or complex (*Fraxinus excelsior* L.) leaves. Needles of coniferous species are particularly effective [61, 62]. Due to their prominent LAI, highly effective pollutant capture is also observed in climbing species, like ivy *Hedera helix* L. [63]. Presently, climbers are often included into green infrastructure in cities as a solution for air purification, and urban heat island (UHI) mitigation.

*Morphological characteristics of leaf surfaces* Generally, the leaf trichomes are considered to improve the efficiency of air purification. Yet, the influence of trichomes is not unequivocal. In numerous observations, the correlation between the presence of trichomes and the amount of PM was not confirmed [57, 58, 64]. It seems to be related rather with the trichome morphology, density, arrangement and chemistry, than just their mere presence, so taking only this feature as a factor may be misleading. Going into details, in some species, like *Alchemilla mollis* (Buser) Rothm. or *Triticum aestivum* L., trichomes form a water-repellent layer [65, 66], whereas their structure in other species is favorable for particle capturing. Another feature which may favor PM capture is foliar roughness [67]. The ridges (at a scale of 1–2 μm) on leaf surfaces were more efficient at accumulating PM, particularly PM_2.5_, compared with the roughness (*P*–*V* distance) of 5–20-μm scale [68]. Yet, similarly to trichomes, the effects of groove presence seems to be elusive [69]. Facing the complexity of leaf surface structures and how they relate to PM capture, it seems reasonable to rather research the comprehensive ability of different plant species for air purification. Such method was introduced by Sgrigna et al. [69]. They proposed Accumulation Index *A*_i_, considering the foliar roughness, trichome presence, stomatal density, and macromorphological traits of leaves: complexity of margins, leaf shape, leaf growth expansion, and the type of foliage (evergreen or deciduous). Save for surface complexity, anatomical structure of needles of coniferous species, specifically, the number and location of resin channels seems to be responsible for trapping xenobiotics [70].

*Cuticle* The presence of wax cuticles is considered to be a factor responsible for a plant’s potential for air purification. Yet, there are many reports that contradict this statement. No statistical correlation between the presence of wax cuticles and air purification abilities was evidenced for 8 tree and 4 shrub species in Poland. Detailed studies indicated such relationship only for two species and a single PM category, i.e. for PM_2.5_ deposited in the wax layer of *Forsythia* × *intermedia* Zabel leaves and coarse particles on leaves of *Tilia cordata* Mill. [71]. Similarly, no such relationship was found for trees in Beijing [57]. Moreover, *Eucalyptus globulus* Labill., despite its well-developed cuticles, had the lowest ability to collect PM among species studied by Freer-Smith et al. [62]. This suggests that the efficiency of PM capture depends rather on the chemical structure of the cuticle and the lipophilic or polar character of the pollutant, than the amount of wax [71, 72]. For example, it may be related to the structure of wax crystals on cuticle surfaces, hydrophilicity of the amorphous waxes, as well as the length (from about 2 to 6 nm) and chemical structure of wax chains, protruding from crystalline regions. High effectiveness of xenobiotic capture of conifer needles is, among others, attributed to the presence of long-chained polyesters [73]. Water permeability of the cuticles, attributed to the amount of polysaccharic strains, may be also involved in a plant’s ability to retain polar compounds and ions. The highest performance of both features, which covers the same group of species, can support this suggestion. According to Riederer and Schreiber [74], the highest permeability of leaf cuticles is observed within deciduous trees in temperate climatic zones. Many of them, like *Betula pendula, Tilia cordata*, *Syringa reticulata* (Blume) H.Hara and *Magnolia denudata* Desr. are also the most efficient in PM capturing [75]. On the other hand, super-hydrophobic character of the mentioned *Eucalyptus globulus* cuticles seems to be the reason of the poor PM deposition. Liang et al. [57] found the relationship between the amount of captured PM and epicuticular wax ultrastructure. Because interfacial area affects the adhesion force, the most beneficial one was the thin wax film of elm *Ulmus pumila* L. in contrast to the dense tubular wax of *Gingko biloba*.

*Phyllosphere* Recent research draws attention on another aspect that can explain the positive correlation between the cuticle thickness and plant immobilization of ambient pollutants, which is the phyllosphere [76]. Leaves and other plant organs are colonized by bacteria, fungi, yeast, and algae. Much has been written regarding the ability of endophytic fungi and bacteria to degrade toxic contaminants, for example PAHs, which are then used as a source of carbon and energy [77, 78], but little is known about ones colonizing the surface of leaves. Wei et al. [79] list, among others, the following bacteria: *Methylobacterium, Sphingomonas, Beijerinckia, Azotobacter, Klebsiella,* and *Stigonema*, and fungi: *Aureobasidium, Cladosporium,* and *Taphrina*, yet their communities may differ according to the plant species, habitat conditions, or even the type of pollutants. Many of them are very efficient in capture and detoxification of air contaminants. Their population in five ornamental species growing on the roadsides of Sri Lanka ranged from 1.28 × 10^3^ to 9.6 × 10^6^ CFU g^−1^ (from total 6.2 × 10^5^ to 8.1 × 10^8^ CFU g^−1^), with the most densely populated phyllosphere of *Ixora chinensis* Lam. Among them *Aspergillus, Cladosporium, Nigrosporium, Trichoderma, Mucor, Penicillium, Pericornia, Humicola, Broomella, Coletritichum, Cylindrocarpon, Acremonium* and *Aeurobacidium*. *Penicillium oxalicum* degrading 80% phenanthrenes and 96% naphthalene, and *Coletritichum siamense* degrading xylene, phenanthrene, naphthalene, and toluene in a range of 52–68.9% occurred to be superior to others [80].

Bacteria are a more frequently studied microbes of the phyllosphere. As Yutthammo et al. [76] reported, the total phyllospheric bacteria of ten ornamental species in Bangkok accounted for 2.31 × 10^3^ to 1.25 × 10^5^ MPN g^−1^, of which the amount of PAH detoxifying bacteria ranged from 1 to 10%. Further studies allowed to classify the PAH degrading bacteria *Wrightia religiosa* (Teijsm. & Binn.) Benth. ex Kurz phyllosphere: *Sphingomonas, Acinetobacter, Mycobacterium*, *Pseudomonas*, and many non-cultivable taxa from. They were effective in the detoxification of both low- and high-molecular-weight compounds. Apart from *Pseudomonas*, PAH-degrading species from different genera, including *Microbacterium*, *Rhizobium*, and *Deinococcus* were identified on *Ixora chinensis* leaves [81]. In *Magnolia grandiflora* L. and *Cedrus deodara* (Roxb. ex D.Don) G.Don phyllosphere Franzetti et al. [82] identified *Hymenobacter, Sphingomonas, Methylobacterium* and *Massilia*, but they were not able to distinguish the exact taxa responsible for the xenobiotic catalysis. The latest findings proved that not a single species, but whole microbial consortia may be responsible for PAH degradation [83], and this may be the reason for these difficulties. However, due to the negative interactions among different bacterial populations, their effectiveness may be limited [84], Yet, the interplay between the plant host and its microbiota seems to be of prominent meaning [85]. The interaction between the plant and the microorganisms is involved in many metabolic processes and include, among others, bio-fertilization, stimulation of growth and development (e.g. bacteria-derived IAA) as well as stress mitigation via antioxidative properties of some bacteria and fungi [86, 87]. Mitigation of plant stress supports stabilization of ambient pollutants, e.g. heavy metals [87], but from an ecological point of view their catabolic potential towards contaminants seems to be of major importance. A transcriptomic analysis confirmed that PAHs initiated the expression of genes encoding enzymes responsible for different stages of xenobiotic degradation [51]. For example, endophytic microbial degradation of PAHs takes place via the cytochrome P450-mediated pathway [88]. Research on whether similar relationships exist in the phyllosphere is in progress [87]. Until recently, the presence of PAH-catabolic enzymes, like naphthalene-1,2-dioxygenase, aryl-alcohol dehydrogenase, catechol-1,2-dioxygenase and catechol-2,3-dioxygenase was confirmed in phyllospheric bacteria [82]. In fungi, the monocyclic aromatic hydrocarbons (MAH) and PAH-degrading ability are often connected with lignin-degrading systems and the presence of lignin peroxidase, manganese peroxidase, and laccase, which is also responsible for wood-decaying. According to Kannangara et al. [89] manganese peroxidase was the key enzyme in naphthalene degradation, while strains of non-lignolytic fungi took part in the degradation of PAH (benzene, toluene, and xylene). Their ability for this process is based on the activity of distinct enzymes, like cellulases and starch-degrading amylases. In contrast to bacteria, PAHs cannot be the only source of energy for fungi. What is more, most of them are not able to degrade high-molecular-weight PAHs. Thus, for full degradation, the complex cooperation between microorganisms from different kingdoms is in some cases pivotal and include exchanging byproducts and metabolites. Moreover, fungal mycelia may act as transport network for bacteria, supporting their chemotropism towards PAHs [51].

Degradation is not the sole pathway of plant-microbiome interplay. Phyllosphere-derived enhancement of plant growth, by plant hormone production, increased nutrient availability (e.g. by production of organic acids), support stress tolerances leading to an increased LAI, therefore the area for PM accumulation [86]. Phyllospheric microorganisms also improve the bioavailability of pollutants by excretion of biosurfactants [51]. On the other hand, the interaction between plants and microorganisms involves positive feedback from plants, which can enhance the degradation abilities of microorganisms. It was observed that some plant products, like salicylic acid, flavonoids, and fatty acids can stimulate the expression of bacterial genes responsible for xenobiotic degradation [51, 88, 90].

##### Factors connected with the environment and city design

The influence of environmental factors on plant-derived air purification harbors diversified features: from the traits connected with PM itself, air circulation, to the relative humidity and temperature [91]. For PM, important features include its concentration, size, and chemical character. The concentration drops down along with the distance from the source of contaminants [92], hence the most beneficial are plants growing close to the site of the PM origin. For the greatest effectiveness, a plant barrier should be placed between the source of pollutants and the exposed people, taking into account the usual wind directions [92]. According to Morakinyo et al. [93], a noticeable drop in PM levels is observed in a certain distance from the barrier, 7–12 m for oblique winds and 8–18 m for perpendicular winds. To achieve dense deposition, it is crucial to create conditions for air circulation through the canopy. The air movement is one of the key factors in PM deposition as it drives particles close to the leaves. A positive correlation between pollutant deposition and wind speed was revealed [94, 95]. Wind acceleration from 3 to 9 m s^−1^ resulted in a significant rise in the deposition velocity and capture efficiency for all examined taxa of trees [61]. Yet, the wind speed, direction, and the rate of foliage penetration may be modified by various factors, including the climate and weather, the height and design of architectural barriers and plants themselves. Three-dimensional geometry of a city is associated with numerous canyons between buildings, noise barriers, and others which act as sinks for turbulence, limiting the contaminant dispersion and leading to the local rise in pollution [96]. For this reason, the proper design of city infrastructure is required. What is more, tall and dense trees forming barriers that are passed above, rather than penetrated, are not desirable [59, 97]. Instead, planting lower trees with loose branches and foliage, and including hedges or other types of lower vegetation beneath the trees is suggested [96]. In open spaces, planting “green spots” with numerous trees and diversified vegetation, spatially separated from the source of pollution, is recommended.

The other aspect of PM capture is the retention of airborne particles. Many airborne pollutants are stored on leaves temporarily. Due to winds, PM is re-suspended in the atmosphere and with rain, it is washed off onto the soil. Greater amounts of airborne pollutants deposited by deciduous species get to the soil with the falling of leaves. Regarding the effects of rainfall on PM removal, Xu et al. [98] indicated the key role of precipitation intensity through increased kinetic energy of drops. It is applied within the intensity not exceeding 30 mm h^−1^. A rise above this value does not substantially increase PM removal [98, 99]. The influence of the duration was much weaker, as PM removal was the strongest at the beginning of a rainfall, i.e. first 2.5 mm of precipitation (up to 30 µg cm^−1^) [98, 99]. The amount of re-suspended particles depends also on the leaf surface complexity and wettability. Wash-off experiments indicated that the rates of PM removal from leaves can differ strongly within the range: from 6.4% of total PM for *Platanus* × *hispanica* to 87.6% for *Olea europea* L. [100]. Generally, coarse particles are washed off at higher rates as they are usually deposited on the leaf surface, whereas fine particles are more likely embedded in the wax layer. To move them from the cuticles, a heavy rain event is required [99]. Similarly, leaf surface adhesiveness, roughness, and more importantly, the stomatal density are connected with the rate of resuspension caused by winds [101]. The other factor controlling the release of PM is wind speed. The amount of released PM increases with wind acceleration, when aerodynamic, vibrational, and mechanical removal forces break the forces of adhesion, i.e. van der Waals, capillary, and electrostatic [101, 102]. These forces are modified by the relative humidity. A high humidity enhances the adhesion due to capillary condensation between the PM and a surface [102]. Regardless of the weather conditions, deciduous species lose deposited pollutants every year in autumn, while contaminant retention of coniferous trees lasts 2–5 years, depending on the longevity of needles of a species.

### The role of green infrastructure in PM reduction

A substantial increase in green coverage and species diversity can be implied by green and blue infrastructure solutions, like green roofs, green walls, and vegetated water retaining systems, as well as urban farming [18, 103, 104]. The significance of green infrastructure in cities is underlined in the newest reports released worldwide: the 2030 EU Biodiversity Strategy [18], and UN Habitat [105] serve as a means of providing/supporting sustainable and inclusive development of urban areas.

#### Green roofs

Green roofs are at present one of the most popular and widespread green technologies, supported by numerous policies or demanded by law worldwide. Their pivotal function in the city is water retention, but their role in climate improvement (mitigation of UHI, CO_2_ sequestration, air pollution removal, and energy savings in buildings) is also appreciated. The key factor influencing their biodiversity and ecological services is the thickness of the substrate layer. The deeper it is, the richer selection of plants can be applied. In extensive roofs, with the thinnest substrate of 2–20 cm [106], usually succulent species such as *Sedum* sp. can thrive. However, they are regarded as non-coherent pollutant removers. For example, the potential of *Sedum album* L. in trapping PM was 0.42 g m^−2^ year^−1^, compared to 3.21 and 1.81 g m^−2^ year^−1^ of *Festuca rubra* L. and *Agrostis stolonifera* L., respectively. Yet, in semiarid areas, this species exhibited a higher rate of PM deposition reaching 29.32 µg cm^−2^ h^−1^, compared to other *Sedum* species (with *S*. *reflexum* L.*,* and *S. palmeri* S.Watson having the lowest effectiveness) and plants, such as *Pittosporum tobira* (Thunb.) W.T.Aiton and *Erigeron karvinskianus* (DC.) Kuntze (1.38 and 1.62 µg cm^−2^ h^−1^ respectively) [107]. Due to potentially more abundant and diversified vegetation, intensive and semi-intensive green roofs are more effective in green roof PM deposition. In a study conducted in Montreal, the GRs covered with *Pinus mugo* var. *pumilio* (Haenke) Zenari on the buildings heated with wood were able to remove 4.00 g m^−2^ of PM_10_ and 1.52 g m^−2^ of PM_2.5_ annually [108]. The ability of green roofs to capture PM is often compared to that of trees. It can be noticed that green roofs are not as efficient as trees, although, in some cases, their PM capture ability can reach values close to those achieved by trees [109]. Yet, it is worth underlining that green roofs are not competitors to trees, but act as their supplement [110]. Their popularity and area that they occupy in cities add substantial improvement to air pollution reduction. For example, according to data from 2015 there were 4 million m^2^ of green roofs in Berlin, 3.148 million m^2^ in Munich and 1.345 million m^2^ in Tokyo. The greatest green roof area per capita belonged to Basel, Switzerland, a town with a population of 175,000 and 1 million m^2^ of green roofs (5.71 m^2^ per inhabitant). These data have changed noticeably as green roofs have continuously been adopted [111].

#### Living walls

Similarly, green walls are facing a growing interest for mitigating air pollution. Depending on the species composition, the LAI of green walls is around 1–2 m^2^ for every m^2^ of wall. In the study of Weerakkody et al. from Stoke-a-Trent (UK), multi-species green walls abated, on average, 122.08 × 10^7^ PM_1_, 8.24 × 10^7^ PM_2.5_ and 4.45 × 10^7^ PM_10_ [112]. However, to acquire a significant purifying effect, their localization should be well-designed [59]. The most pronounced influence can be observed when green walls are installed in small, closed spaces, such as street canyons. Wind speed and canyon geometry determine the PM concentration in street canyons. At the most favorable aspect ratio of *height/weight* (*h/w*) = 2 and low (0.5 m s^−1^) wind speed, green walls may enhance the sink for pollutants and contribute to the drop of PM_10_ concentration in street canyons by 60% [94]. It may also positively affect the PM_2.5_ concentration behind the wall at the pedestrian height (1.4 m), but increase it over the wall [93]. The distance of worsened air quality rises with increasing heights of green walls. Facing such an occurrence, it is not recommended to install green walls close to high buildings. Following these phenomena, modeling studies of particular cases should be conducted, taking into consideration numerous factors such as plant species, canopy volume, wind speed, and direction, as well as the urban geometry [93, 94].

Recently, a great progress has been done with green walls towards their utilization in PM capture. To increase the purifying potential of plants air flow through canopy and growing medium with plant rhizosphere is supported by active mechanical ventilation. Generally, active green wall technology, called also botanical filtration, is known for its efficiency in VOCs and PM abatement indoors [113–115], however it is worth noticing, that considerable amount of PM indoors is of outdoor origin [116]. According to Irga et al. [117], the single-pass removal efficiency (SPRE) of ambient indoor VOCs, which can reach 70% and does not depend on plant species, whereas species of plant determines single VOC removal. *Chlorophytum orchidastrum* Lindl. and *Schefflera arboricola* (Hayata) Merr. were the most efficient in ethyl acetate removal, whereas *Nematanthus glabra* in benzene. *Chlorophytum orchidastrum* also occurred to be an effective purifier of PM, yet the most PM of all fractions was captured by *Nephrolepis exaltata bostoniensis* (e.g. SPRE for PM_5-10_ reached 92.46%) [118]. Recent studies dealt also with application of active green walls for ambient PM removal as well [119]. The results confirmed some reduction of PM after Black Summer in Sydney in 2020 and suggest further research on the use of botanical filtration outdoors.

Except for numerous reports about the use of botanical filtration indoors, there is still too little knowledge about them. None of the studies, to our best knowledge, compares effectiveness of pollution abatement of passive and active green walls [120]. The mechanism which is mainly involved in air purification is also unclear, although in some papers rhizosphere activity is considered to be more effective in PM and VOCs removal [19, 120–122]. Increased area of capturing surface, abundant microflora connected with plant taxa and growing medium and root exudates are involved in rhizosphere predominance [117, 123]. Hence, the preservation of microbial activity is one of the main concerns for air pollution abatement. It may be sustained by plant species and medium selection, or continuous inoculation with new bacteria or fungi, e.g. with irrigation [120]. Another consideration should be given to growing media. They should maintain both, plant growth and microbial abundance and diversity. Very often growing media themselves can reduce air contaminants. Porous materials with increased surface, like activated carbon, zeolite, silica gel and polymers are the most beneficial for pollution capture [124, 125].

#### Water reservoirs

Wind speed seems to play a crucial role in PM removal by wetlands. Rivers and water reservoirs, as parts of blue infrastructure, seem to be undervalued in terms of their pollutant removal capacity. Apart from dry deposition on the surface of water [126] wetland plant species are involved in PM deposition. Due to a lower LAI, their rates of purifying the air are lower than those in a forest, yet the deposition velocities may be almost the same in these two types of vegetation. This indicates the high potential of wetland plants, such as *Phragmites australis* (Cav.) Trin. ex Steud.*, Scirpus Validus* Vahl, and *Iris* spp. for both fine and coarse particle removal [127]. Another study indicated the high PM capture efficiency of *Phragmites australis* (42.14 1.30 μg cm^−2^ of PM_10_ and 11.30 μg cm^−2^ of PM_2.5_). These rates were respectively more than 30 and 3 times higher than the lowest such efficiency observed in *Lythrum salicaria* L. (4.58 and 1.30 μg cm^−2^, respectively) [128]. Yan et al. [99] demonstrated an even higher potential of *Typha orientalis* C.Presl for PM capture.

#### Urban farming

Another trend in green infrastructure is urban horticulture and agriculture. It is a response to the growing demand of fresh and local foods in order to shorten the food chain. It is perfectly included into the idea of a circular city, where waste organic matter and wastewater is reused. It involves a number of advanced sustainable green technologies, such as hydro- and aeroponics, vertical farming, rooftop gardens, as well as traditional forms of growing food like private kitchen gardens and allotments. A new, but more and more popular form of food production are community gardens which aim at integrating local societies.

To supply food, urban food production must solve the problem of environmental pollution. PM suspended in the air is exceptionally hazardous as they contain harmful heavy metals and PAHs. They can enter plants directly from the air or indirectly from the soil, after its deposition on soil surface.

Metals do not undergo decomposition so they accumulate in soils at high levels [129, 130]. From soils, they enter plants via the roots and accumulate in plant tissues. The other pathway, through leaves and stems, is less common. Their risk to human health comes from ingesting contaminated food and, to a lesser extent, inhalation of metals suspended in the air and contacting these metals through the skin [131]. In the human body, they cause multiple injuries to the liver, kidneys, brain, bones, lungs, and impair fertility, depending on the element. Some of them (As, Cd, Cr, Ni, Pb, Zn, and V) are also responsible for various types of cancer [132, 133]. According to Romanian studies, these toxic effects may contribute to decrease in the human life expectancy by 9–10 years [134]. An important issue is also processed food made of polluted crops, in which metal contamination can condense.

Heavy metal load on leaves takes place via the same mechanisms as other PM: by being captured on the leaf surface and encapsulated in the cuticle. Metals trapped on leaf surfaces may be removed in great part by washing [135], so the main risk from metals comes mostly from penetration of leaves in two ways [117, 123]: hydrophilic compounds get to leaf tissue through the stomata or pores in the cuticle wax layer, like cracks, ectodesmata, and aqueous pores, while lipophilic ones penetrate the cuticle. This phenomenon highly depends on the metal speciation. For example, Pb in PM can exist in the form of PbS, PbSO_4_, PbSO_4_-PbO, a-PbO, and PbO [136, 137]. Furthermore, heavy metals themselves can interact with the cuticle and change its structure and decrease its permeability [138]. The trapped metal/metalloids are also exposed to humidity, high temperature, solar radiation, and microbiological activity of the phyllosphere which can change their accessibility. Yet not all the processes connected with metals entering plants through leaves are revealed in contrast to soil-derived pollution. In terms of transport, it is suspected that air-derived metals and metalloids are more reluctant to move than those coming from the soil. It may by caused by the fact, corroborated for Pb, Cu, Zn and Mn, that they express a high binding capacity to cellulose and are immobilized in the cell walls of the leaf tissue [138]. On the other hand, there are metals with a high mobility in plants. Research of Natasha et al. [139] indicated that arsenic, after foliar application, was transported to the roots, contrary to lead, which was only accumulated in the leaves.

The potential of a plant species to accumulate metal or metalloids seems to be of the greatest importance in airborne pollution ingestion via vegetables [119, 126], yet there exists little research to classify crop species as tolerant or hyperaccumulators towards a specific pollutant [138]. As ingesting is the main pathway of entering the human body, the quality of edible parts of crop plants is of the greatest concern. Due to PM capture and high immobilization of metal and metalloids, leafy vegetables are the least secure for cultivation in urban areas [139, 140] (Table 1). Previous experiments give evidence that lettuce *Lactuca sativa* L. collects alleviated amounts of Al, As, Mn, Pb and Cu, *Spinacia oleracea* L.—As, Cd, Zn and Fe [124, 127], *Brassica oleracea* L.—Zn and Sb [132], while *Chrysanthemum coronarium* L.—Cr [141]. Generally, the health risk assessment which includes numerous parameters such as the hazard index (HI) for adult and children, hazard quotient (HQ), estimated daily intake (EDI), maximum daily intake (MDI), lifetime cancer risk (ILTCR), and total hazard quotient (THQ). This methodology allows assessments of the risk of a plant species and a metal pollutant, and indicates, for example, that hazard index of Cd, Zn, Sb in case of *Brassica oleracea* (0.924 for adults and 1.801 for children) is almost 20 times higher than that for *Spinacia oleracea* (0.053 and 0.103, respectively) [142]. The limit value of HI is 1.0, which is exceeded in the case of *B. oleracea* for children.

**Table 1:** The concentration of heavy metals (mg kg^−1^ of dry weight) on edible organs vegetables grown in different world cities

Edible organ	Species	Country	References	Pb	Cd	Cu	Ni	Cr	Zn
Leaf	Lettuce *Lactuca sativa* L.	France	[136]	355.0					
		France	[206]	122.0	1.7	6.8			29.1
		South Korea	[141]	0.70–1.26	0–0.08		6.54–8.36	0.36–0.40	49.6–62.2
		Romania	[134]	68.3–118	4.9–6.7		11.8–13.8		121.0–301.0
		Benin	[207]	77.1–77.8	0.3	3.8–7.9			56.4–107.8
		Ecuador	[208]	0–1.18	0–0.56				
	White cabbage *Brassica oleracea subsp. capitata *(L.) *Metzg*	Germany	[209]	2.6	0.41	6.6	1.0	0.81	46.5
		Benin	[207]	52.6–78.9	0–0.1	1.8–3.9	–	–	44.1–85.4
		China	[210]	1.71^a^	0.13^a^	1.40^a^	–	–	5.95^a^
	Chinese cabbage *Brassica rapa *var*. pekinensis (*Lour*.) Hanelt*	China	[210]	1.17^a^	0.34^a^	1.69^a^	–	–	12.4^a^
	Pakchoi *Brassica rapa *var*. chinensis *(L.)* Kitam*	China	[210]	2.79^a^	1.2^a^	3.07^a^	–	–	14.3^a^
	Leek *Allium ampeloprasum* L.	China	[210]	4.03^a^	0.39^a^	2.17^a^	–	–	8.63^a^
	Persley *Petroselinum crispum* (Mill.) Fuss	France	[206]	298.7	0.8	4.4	–	–	25.4
	Spinach *Spinacia oleracea* L.	South Korea	[141]	0.59–1.16	0		7.17–50.9	0.11–0.36	91.53–122.1
Fruit	Tomato *Lycopersicon esculentum* Mill	Germany	[209]	0.1–6.7	0.01–0.79	3.5–16.0	0.03–0.70	0.11–0.63	15.8–84.7
		Ecuador	[208]	0–2.33	0–0.66				
		China	[210]	0.72^a^	0.39^a^	1.39^a^			3.48^a^
	Pepper *Capsicum annuum* L.	China	[210]	1.94^a^	0.23^a^	2.0^a^			4.3
	Green beans *Phaseolus vulgaris* L.	Germany	[209]	0.1–3.5	0–0.04	3.5–10.5	0.27–1.3	0.08–0.46	32.4–44.2
Root	Carrot *Daucus carota* L.	Germany	[209]	1.3–28.5	5.4–23.2	5.4–23.2	0.07–0.47	0.1–2.39	23.3–122.8
		Belgium	[211]	0.9–9.2^a^	5.6–10.9^a^				
	Celery *Apium graveolens* var. *Rapaceum* (Mill.) Poir	Belgium	[211]	1.6–21.0^a^		10.9–40.1^a^			
	Radish *Raphanus sativus* L.	China	[210]	1.01^a^	0.22^a^	0.96^a^			3.57^a^
Stem	Kohlrabi *Brassica oleracea* var. *gongylodes* L.	Germany	[209]	0.1–3.1	0.03–0.15	3.2–11.6	0–0.91	0.07–0.54	20.6–50.3

^a^Concentration in mg kg^−1^of fresh weight

Considering the toxicity and potential vulnerability of organisms, As, Cd, Cr, Pb, and Hg are believed to be the most hazardous [138]. Yet, numerous studies from Europe and the USA show that growing vegetables and fruits in cities can be safe, unless the cultures are in the vicinity of mines, heavy-traffic roads, and industrial companies as well as arable land being fertilized, at present or in the past, with sewage water or sludge. A study conducted by Noh et al. [141] suggest that washing of contaminated vegetables also helps to keep the level of PM below the limit value.

There are many routes via which PAHs can enter the human body, but ingestion is the most common and the most profound [50]. Contrary to heavy metals, intake of PAHs from fruits and vegetables is estimated to be 5–10%, while their global consumption is around 30% of all food products. The rest comes mainly from animal and oil products, much of them during food preparation and storage. Some PAHs can be produced by plants [143, 144]. Due to the high risk to PAHs present to the human and animal health, especially their carcinogenicity and mutagenicity, their presence in horticultural products draws attention in cultivation in polluted environments.

The biggest source of PAHs is the incomplete combustion of fossil and organic fuels. In a city, we can find numerous such sources: high traffic, industry, and stoves in residential areas [104]. For that reason, cultivation of vegetable and fruit crops in urban environments results in a higher PAH absorbance compared to that in rural areas [50, 145]. As the above-ground parts of vegetables are polluted more than the underground parts, aerial exposition seems to be the main route of PAHs in urban crops [146]. Contrary to soil contamination, out of 16 PAHs which are usually analyzed, low-weight PAHs are usually accumulated on leaves. It is connected with their volatile and hydrophilic character [37].

The accumulation of PAHs in horticultural crops depends on the species, and even on the variety [50]. As in the case of HMs, leafy vegetables like lettuce and Chinese cabbage retain the greatest amount of PAHs [130, 132], yet, according to Jia et al. [147], the highest amount of ∑PAHs was detected in romaine lettuce *Lactuca sativa* var. *longifolia*. The least hazardous vegetable for consumption seems to be cabbage [130, 132, 133]. In fruits, root vegetables, and fruiting vegetables the majority of accumulated PAHs is immobilized in peels, so it is possible to diminish the amount of ingested PAHs by removing the peels [148].

#### The effects of urban vegetation on PM reduction

The reduction of PM levels in the air was corroborated in numerous papers, indicating a negative correlation between the total green area and PM concentrations [91, 149–151]. Yet, most of them focuses on the role of trees in this phenomenon, although herbaceous plants substantially support the capture potential of trees [152]. McPherson et al. [153] estimates the amount of PM_10_ accumulated in trees in Chicago (USA) to be 212 t per year. According to Nowak et al. [154] in the whole USA, this value for all airborne particles can reach 711,000 t year^−1^. This means about 0.2–1.0% reduction in the PM levels. In Manchester (UK) city center, trees can store 0.24 t of PM_10_ a year i.e. about 2.3% of PM_10_ in the area [107]. 850–2100 t of PM_10_ is removed yearly in London [155], an equivalent of 0.7–1.4%. A high reduction in PM_10_ was also noted in Santiago de Chile [156]. In areas with high pollution and 26% vegetation cover, usually inhabited by low-income citizens, 1.6% PM level reduction was achieved with trees and an additional 1.1% with shrubs. Simulations give more promising results: data from London suggest that the maximum green coverage rate (56% of the area) can locally adsorb 26% of suspended PM_10_, which would amount to 200 t per year [157].

There are conditions that limit PM capture. Some are related to the city design, i.e. street canyons with dense tree canopies [40, 94]. Furthermore, in northern regions, vegetation seems to have a limited effect on PM capture [158].

### Methods of increasing the PM capture potential of plants

#### Species selection according to their PM capture potential

The differences in PM accumulation capacity among tree species can be 10 to 20-fold [159], thus thorough an appropriate choice of species, a considerable improvement of air purification may be achieved. In Tehran (Iran), the effect of replacing trees with species with a higher potential for xenobiotic capture can be estimated for 14.4% (CO, NO_2_, O_3_, SO_2_ and PM_2.5_), while the improvement in air the PM_2.5_ level is valued for 10.69% [160]. Moreover, a 40% improvement can be afforded by planting additional 150,000 trees with a high PM capture potential.

Many studies have analyzed the PM capture potential of trees, especially species originating from temperate climates from Europe, Asia, and North America [161]. In terms of PM capture, the most frequently analyzed tree taxa include *Acer, Fraxinus, Pinus, Prunus, Populus, Quercus, Ulmus, Tilia, Platanus* and *Betula* genera. Yet, the differences in the experimental design and conditions with the dominating role of climate and seasons make the comparison of results very difficult. Another problem is the variety of values taken into account: foliar retention Cp (usually expressed in μg cm^−2^) (Table 2) [71, 162], deposition velocities (cm s^−1^) [62, 163], but also daily/yearly retention efficiency (mg m^−2^ d^−1^) [164, 165], number density (particles mm^−2^) [53, 166], and saturation isothermal remanent magnetization (SIRM) (µA) [167]. Furthermore, results of PM abatement may differ according to the analytical method used. For instance, in the study by Sgrigna et al. [69] the capture depositions analyzed by vacuum filtration and energy-dispersive X-ray spectroscopy methods were consistent only for six tree species out of twelve. Distinct results, among others, were achieved in the case of *Platanus acerifolia*. It expressed a high PM capture efficiency by SEM/EDX, but a low PM capture efficiency when analyzed by F/V method. After the use of the same method Dzierzanowski et al. [71] also qualified *P. acerifolia* as a lowly effective purifier, whereas in other publications it was also mentioned among trees with the highest potential for PM capture [166, 168]. Such discrepancies were revealed for other species, like *Tilia cordata* [71, 162] and *Styphnolobium japonicum* (L.) Schott [68, 169, 170]. According to Sgrigna et al. [69], this phenomenon may result from the lack of specific solvents to extract all the particles from the different cuticle layers. Contrary to this, consistent results exist in the case of species with a low PM capture potential, such as *Ginkgo biloba*, *Ligustrum lucidum* W.T.Aiton, *Ailanthus altissima* (Mill.) Swingle and *Eucalyptus* sp. [149, 151, 171, 172]. Deposition data are supported by deposition velocities, with the lowest value for *Eucalyptus globulus*—0.04 cm s^−1^, 0.19 cm s^−1^ for *Acer campestre* L. and *A. pseudoplatanus* L., 0.42 cm s^−1^ *F. excelsior* and the highest for *Pinus nigra* J.F.Arnold—6.84 cm s^−1^ [173].

**Table 2:** List of studies dealing with PM accumulation on tree leaves in different world cities (average values, in µg cm^−2^)

	Study site	Examined taxa	Taxa with the highest capture	Cp	Taxa with the lowest capture	Cp	References
	Wind-tunnel experiment	*Olea europaea, Platanus orientalis, Quercus cerris, Q. ilex, Tilia cordata*	*Tilia cordata* *Quercus cerris*	1.5 0.8	*Olea europaea*	0.2	[100]
Africa	Egypt Sohag	*Albizia lebbek, Bauhinia variegata, Bougainvilla glabra, Casuarina equisetifolia, Cassia glauca, Dalbergia sissoo, Eucalyptus rostrata, Ficus carica, Ficus elastica, Ficus nitida, Ficus platyphylla, Ficus religiosa, Ipomoea cordata, Jacaranda acutifolia, Khaya sengalensis, Luffa sp., Mangifra indica, Morus alba, Morus nigra, Nerium oleander, Plumeria alba, Poinciana regia, Psidium guajava, Thuja orientalis, Tipuana tipu, Vitis sp., Ziziphus spina christi*	*Ficus carica,* *Thuja orientalis,* *Morus nigra*	31.7 27.0 21.5	*Tipuana tipu,* *Dalbergia sissoo,* *Cassia glauca*	4.4 5.2 5.6	[212]
Asia	Beijing, China	*Ailanthus altissima, Amygdalus trilobar, Euonymus japonicus, Fraxinus pennsylvanica, Ginkgo biloba, Lonicera maackii, Magnolia denudata, Pinus armandi, Pinus tabulaeformis., Platanus acerifolia, Platycladus orientalis, Populus tomentosa, Prunus cerasifera, Rhus typhina, Salix matsudana, Sophora japonica, Ulmus pumila*	*Platycladus orientalis,* *Pinus armandi*	156.0 156.0	*Sophora japonica,* *Fraxinus pennsylvanica,* *Populus tomentosa,*	⁓ 26.0^a^ ⁓ 27.0^a^ ⁓ 28.0^a^	[172]
	China Beijing	*24 trees, incl.: Aesculus chinensis, Ginkgo biloba, Koelreuteria paniculata, Salix matsudana, Tilia tuan, Toona sinensis* *11 shrubs, incl.: Amygdalus triloba, Chimonanthus praecox, Euonymus japonicus, Paeonia suffruticosa, Prunus cerasifera, Weigela florida*	*Platanus acerifolia, Quercus variabilis, Cephalotaxus sinensis,* *Broussonetia papyrifera*	160.9 ⁓ 160.0^a^ ⁓ 143.0^a^ 127.0	*Ailanthus altissima, Sophora japonica,* *Fraxinus mandschurica*	⁓ 14.0^a^ ⁓ 18.0^a^ ⁓ 36.0^a^	[168]
	China Hangzhou	*Amygdalus persica, Eucommia ulmoides, Ginkgo biloba, Magnolia denudata, Metasequoia glyptrostroboides, Osmanthus fragrans, Platanus acerifolia, Sapindus mukorossi, Schima superba, Ulmus parvifolia*	*Platanus acerifolia,* *Sapindus mukorossi, Metasequoia glyptrostroboides*	⁓ 118.0^a^ ⁓ 102.0^a^ ⁓ 101.0^a^	*Schima superba,* *Ginkgo biloba*	⁓ 34.0^a^ ⁓ 35.0^a^	[166]
	China Kunming City	*Celtis kunmingensis, Cinnamomum camphora, Cinnamomum japonicum, Euonymus japonica, Ligustrun lucidum, Loropetalum chinense, Magnolia grandiflora, Osmanthus fragrans, Photinia glomerata, Pinus tabuliformis, Platanus acerifolia, Prunus cerasifera, Prunus majestica, Rhododendron pulchrum*	*Magnolia grandiflora, Platanus acerifolia, Cinnamomum japonicum*	4.1 3.6 2.6	*Cinnamomum camphora,* *Prunus majestica, Ligustrum lucidum*	0.8 1.3 1.5	[171]
	India Odisha	*Albizia lebbeck, Alstonia scholaris, Anthocephalus indicus, Bougainvillea spectabilis, Cassia auriculata, Cassia siamea, Caesalpinea pulcherima, Delonix regia, Ficus religiosa, Lagerstroemia speciosa, Mimusops elengi, Peltophorum inerme, Swietenia mahagoni, Tabebuia aurea, Thevetia nerifolia*	*Alstonia scholaris, Lagerstroemia speciosa*	1352.0 1300.0	*Delonix regia* *Caesalpinea pulcherima*	137.0 179.0	[213]
	Seoul South Korea	*Ginkgo biloba, Pinus densiflora, Platanus occidentalis, Prunus yedoensis, Zelkova serrata*	*Zelkova serrata*	1.4–3.0	*Ginkgo biloba*	0.1–0.4	[54]
Australia	Australia Sydney	*Acacia linifolia, Allocasuarina littoralis, Acacia longifolia, Acacia parramattensis, Banksia integrifolia, Banksia spinulosa, Callistemon rigidus, Dodonaea triquetra, Elaeocarpus reticulatus, Eucalyptus spp., Hakea salicifolia, Hakea sericea, Melaleuca styphelioides, Persoonia levis, Syzygium australe, Westringia fruticosa*	*Westringia fruticosa,* *Banksia integrifolia, Melaleuca styphelioides*	⁓ 1200.0^a^ ⁓ 950.0^a^ ⁓ 750.0^a^	*Persoonia levis,* *Eucalyptus sp.,* *Acacia longifolia*	⁓ 100.0^a^ ⁓ 200.0^a^ ⁓ 300.0^a^	[170]
	Australia Wollogong	*Brachychiton acerifolius, Eucalyptus ovata, Notelaea longifolia, Pittosporum undulatum*	*Eucalyptus ovata*	100.2	*Brachychiton acerifolius*	77.9	[214]
Europe	Italy Terni	*Acer saccharinum, Catalpa bignonioides, Platanus acerifolia, Cedrus atlantica, Celtis australis, Magnolia grandiflora, Platanus acerifolia, Populus nigra, Populus tremula, Prunus cerasifera, Quercus pubescens, Robinia pseudoacacia, Tilia cordata*	By V/F method: *Tilia cordata* *Prunus cerasifera* *Pinus nigra* by SEM/EDX method: *Platanus acerifolia* *Prunus cerasifera* *Acer saccharinum*	9.7 7.0 6.0 15.6 14.9 12.3	By V/F method: *Populus tremula* *Catalpa bignonioides Platanus acerifolia* *Tilia cordata* *Robinia pseudoacacia,* *Catalpa bignonioides*	1.2 2.2 2.6 1.9 2.4 2.7	[69]
	Norway Stavanger Poland Pęchcin (nursery)	22 trees and 25 shrubs, incl.: *Berberis thunbergii, Carpinus betulus, Fagus silvatica, Popolus tremula, Quercus robur, Robinia pseudoacacia, Symphoricarpus albus, Ulmus glabra*	*Pinus sylvestris* *Pinus mugo,* *Betula pendula,* *Betula pendula,* *Hydrangea arborescens* ‘Anabelle’ *Sorbus intermedia*	55.0 ⁓ 52.0^a^ ⁓ 28.0^a^ 38.4 ⁓ 21.0^a^ ⁓ 16.0^a^	*Acer platanoides,* *Prunus avium,* *Prunus leurocerasus* *Robinia pseudoacacia* *Tilia cordata* *Acer pseudoplatanus*	⁓ 6.0^a^ ⁓ 10.0^a^ ⁓ 15.0^a^ ⁓ 7.0^a^ ⁓ 8.0^a^ ⁓ 9.0^a^	[159]
	Warsaw Poland	Trees: *Acer campestre*, *Fraxinus excelsior*, *Platanus acerifolia*, *Tilia cordata*, Shrubs: *Forsythia* × *intermedia*, *Physocarpus opulifolius, Spiraea japonica*, Climber: *Hedera helix*	*Spiraea japonica,* *Tilia cordata*	⁓ 26.0^a^ ⁓ 21.0^a^	*Platanus acerifolia*	⁓ 13.0^a^	[71]
	Poland Katowice	Trees: *Thuja occidentalis, Quercus rubra, Tilia cordata, Prunus cerasifera, Robinia pseudoacacia, Acer pseudoplatanus, Betula pendula* ‘Youngii’*, Platanus acerifolia,* Shrubs: *Crataegus monogyna, Forsythia* × *intermedia, Ligustrum vulgare,* Vines: *Hedera helix, Parthenocissus quinquefolia, Parthenocissus tricuspidata*	*Prunus cerasifera,* *Tilia cordata* *Platanus acerifolia*	37.42 30.78 28.80	*Hedera helix,* *Thuja occidentalis,*	10.94 12.12	[215]
	Poland Pęchcin (nursery)	*Catalpa bignonioides, Corylus colurna., Fraxinus pennsylvanica, Ginkgo biloba., Platanus acerifolia, Quercus rubra, Tilia tomentosa* ‘Brabant’*)* and six shrub species: *Acer tataricum* subsp*. ginnala, Sambucus nigra, Sorbaria sorbifolia, Spiraea japonica, Syringa meyeri* ‘Palibin’*, Viburnum lantana*	*Syringa meyeri* ‘Palibin’*, Sorbaria sorbifolia,* *Fraxinus pennsylvanica*	32.1 25.7 20.6	*Catalpa bignonioides, Quercus rubra,* *Platanus acerifolia*	7.5 9.7 12.8	[162]
South America	Chile Santiago	*Ligustrum lucidum, Nerium oleander, Pittosporum tobira*	*Nerium oleander*	4.2–13.4	*Ligustrum lucidum*	2.0–5.9	[164]

^a^Data from graphs, approximate

Although numerous studies confirm PM abatement by herbaceous plants indoors [113, 118, 174], limited studies have been involved in the purification potential of herbaceous species in cities. Available records give evidence that these plants are less potent purifiers, but their ubiquitous appearance can substantially contribute to PM reduction. Out of twenty species growing in vertical gardens in the UK, non-woody plants accumulated less particles than shrubs did [112]. The best herbaceous accumulators occurred to be *Thymus vulgaris* L. and *Carex caryophyllea* Lat. Their leaf features that bear resemblance to those of trees seem to govern their ability to immobilize PM, yet the plant architecture also partakes in it—plants with tall and leafy shoots are considered to be the best purifiers [152]. Among European synanthropic plants, *Polygonum aviculare* L. and *Sysimbrium loeselii* (L.) Rich. are the most potent in PM retention, whereas *Festuca rubra* is the least effective [152] (Table 3). Another type of herbaceous vegetation is grasslands. Grasslands, including recreational and representative areas with mown lawn, but also rough grasslands occupy a huge percentage of city areas. In Sweden it accounted for 15–32% of the urban area [175]. In China approximately 40,000 ha of lawn are established every year [176]. Due to such a broad cover, grasses substantially partake in the PM abatement, even if they collect about 30% of PM kept by the forest [177]. Cowherd et al. [178] gave evidence for the distinct potential of low, intensively mown lawn, which abated less than 10% of PM_10_ and tall grasses which retained about 35–45%, compared to 45–67% of PM_10_ retained by cedar forests. Modern trends of increasing biodiversity of urban grasslands and decreasing costs of lawn maintenance by seldomly mowing [179] may contribute to a substantial air quality improvement.

**Table 3:** Accumulation of particulate matter on European herbaceous plant leaves (particle × mm^−1^) in street corridors [152]

Species	Mean	Max
*Achillea millefolium*	212.5	4233.3
*Artemisia vulgaris*	119.2	1054.6
*Berteroa incana*	172.0	2372.2
*Chenopodium album*	219.4	3210.2
*Convolvulus arvensis*	249.3	1391.7
*Elytrigia repens*	59.3	629.6
*Erodium cicutarium*	91.3	1164.8
*Festuca rubra*	13.;8	74.4
*Galinsoga parviflora*	134.7	1615.7
*Lolium perenne*	79.7	676.8
*Plantago lanceolata*	52.0	391.7
*Poa pratensis*	56.4	336.1
*Polygonum aviculare*	454.9	4371.3
*Sisymbrium loeselii*	546.0	2488.9
*Taraxacum officinale*	69.5	813.9
*Trifolium repens*	38.6	150.0

Apart from total deposition, each of the species expresses different potential to various aerosols. It seems reasonable to adjust vegetation to the local PM composition. For example, *Quercus ilex* L., which is regarded a weak purifier, can be used more effectively for Pb capture than *P. acerifolia* and *T. cordata* [100], which are otherwise considered efficient PM depositors. *Sorbus aucuparia* L. occurred to be efficient in polychlorinated biphenyls accumulation [180]. According to Fellet et al. [181] *Viburnum lucidum* Mill. occurred to be a weak collector of ƩPAHs, but it accumulated the greatest amounts of anthracene.

As biogenic emissions are about 9 times greater than anthropogenic ones, the potential of trees to emit BVOCs should be taken into consideration. The major part, i.e. 35–40% of SOA formation belongs to monoterpenes, 15–32% to isoprene and 10% to sesquiterpenes [26]. For this reason, many papers suggest planting low-BVOC-emitting trees in urban environments [182, 183]. Fitzky et al. [184] characterizes tree species according to their potential emission rates (Table 4):

**Table 4:** Classifying of VOC emitting plant species (measured at 30 °C leaf temperature and 1,000 µmol m^−2^ s^−1^ PPFD) [184]

	Concentration (μg g DW^−1^ h^−1^)		
	Low	Medium	High
Isoprene	< 10.0	10.1–30.0	> 30.1
Monoterpenes	< 2	2.1–5.0	> 5.1
Sesquiterpenes	< 0.5	0.6–1.0	> 1.1
Oxygenated VOCs	< 2	2.1–5.0	> 5.1

Among species cultivated in Europe, the authors mention *Quercus robur* L., *Q. pubescens* Willd., *Populus nigra* L. and *P. tremula* L. as high isoprene emitters, as well as *Fagus silvatica* L. as a high monoterpene emitter. On the other hand, *Acer platanoides* L., *Ulmus minor* Mill., *Fraxinus excelsior* L., and *Tilia platyphyllos* Scop. are considered low BVOC emitters. The genus *Quercus* was also responsible for high emissions of isoprene in Japan, whereas *Cryptomeria japonica* was the greatest producer of mono- and sesquirterpenes [182]. Low emitters of BVOC among tropical trees in Santiago de Chile were *Schinus mole* L., *Quillaja saponaria* Poir. and *Quercus suber* L. [185]. In studies conducted in India, *Ceiba petendra* L., *Cinnamomum zeylanicum* Bereyn., *Azadirachta indica* A.Juss., *Melia azedarach* L., *Chukrasia tabularis* A.Juss., *Terminalia arauna* Roxb. and *T. bellirica* Roxb. expressed low isoprene emission rates, whereas *Dalbergia sissoo* Linn., *Ficus religiosa* L. and *F. infectoria* Willd, emitted high levels of isoprene [186].

#### Increased biodiversity

Loss of biodiversity is one of the major threats to the environment in the contemporary world. Besides the green coverage rate, this phenomenon is assigned to have a fundamental importance in PM reduction. The poor species composition of flora has a negative effect on ecosystem services (ES) of plant communities, like UHI mitigation, water retention potential, landscape connectivity [187] as well as air pollution [75, 134]. The positive correlation between the biodiversity and net primary productivity (NPP) [188], and its relationship with the LAI [189, 190] are involved in this phenomenon. The general trend shows that functional traits of plants are more meaningful for the ES than species richness [191], and seems to be also responsible for the ability of flora to capture PM. Manes et al. [192, 193] proved such a relationship between the biodiversity and PM_10_ removal in Italian cities. The mean values of PM_10_ deposition per m^2^ of leaves for various physiognomic-structural vegetation categories (PSVCs) may differ from 0.75 g m^−2^ in beech woods to 1.93 g m^−2^ in holm oak woods [193]. The authors indicated that yearly PM sequestration of highly biodiverse PSVCs, like holm oak woods, relates to their higher LAI or the share of evergreen foliage (of Mediterranean maquis), which can retain PM throughout the year. Another issue is the emission of BVOCs. A highly complex species richness and vertical diversity through the forest canopy is required to prevent leaves from overheating and increasing BVOC production [194].

Biodiversity provides air pollution abatement via various pathways. Plant diversity supports plant community resilience and diminishes plant stresses in difficult urban conditions, via a more effective use of resources. It also improves the water availability in soils by increasing the water retention and microflora activity. All these allow plants to maintain their water balance and stomatal conductance, which influence their capacity to remove airborne PM. Furthermore, improved condition of plants increases their tolerance to pollution stresses [195]. What is more, abundant vegetation mitigates climate, leading to reduced BVOC emissions and, in response, diminished levels of secondary PM. Additional values is included to this effect through enhanced carbon sequestration.

#### Transgenic plants

High hopes for improvement in the air purification is put into transgenic plants. The potential of genetically modified plants for degradation of both organic and inorganic (mainly metal, metalloid) pollutants has been widely confirmed in vitro [196]. A few classes of organic xenobiotics can be degraded. They include solvents, explosives, PAHs, PCBs, as well as pesticides and herbicides [197]. Genetic engineering of plants in order to improve their phytoremediation of pollutants involves usually three protocols: over-expression of plant genes engaged in the degradation of organic xenobiotics, transfer of microbial genes, and inoculation of plants with endophytic microorganisms with xenobiotic-degrading potential [198, 199]. Until today, different plant species were subjected to genetic modifications to enable their usage in environmental detoxification, with a special focus on *Arabidopsis thaliana* (L.) Heynh., tobacco *Nicotiana tabacum* L., tomato *Lycopersicon esculentum* Mill., rice *Oryza* L., alfalfa *Medicago sativa* L. and various species of cabbage and poplar [199]. Almost all studies in situ were focused on soil and water contaminants removed by the rhizosphere, while the studies on air pollution reduction are scarce. What is more, most of them also relate to the rhizospheric potential for pollution reduction. The first study on the possibility of reducing ambient air pollution with transgenic plants was conducted by Doty et al. [200]. They developed transgenic poplar (*Populus tremula* L. x *Populus alba* L.) by overexpressing cytochrome 450 2E1, an enzyme involved in the metabolism of hexachlorocyclohexane (HCH) in plants. The cuttings of transgenic poplar achieved about tenfold removal rate of low-weight hydrocarbons (benzene, chloroform and trichloroethylene) from the air, compared to the control. The possibility of degrading high-weight PAHs and other organic pollutants seems to be within reach in the near future. Poplars of different species seem to be a key target for genetic manipulations for pollution abatement purposes, due to their remedial capacity of miscellaneous organic and inorganic pollutants, high effectiveness of photosynthesis, intensive growth potential, and ease of genetic manipulations [201].

## Conclusions

A large progress has been made in using greenery in air pollution reduction. However, there still exist big gaps in our knowledge about this phenomenon. One of the biggest problems is the lack of uniform scales for the amount of captured pollutants, which makes a comprehensive comparison of the most effective species for air pollution reduction difficult. We also still cannot be sure about the traits of leaves which improve PM capture. In spite of this, there is an agreement regarding the benefits of green infrastructure in cities, which is locally expected to reduce the ambient PM_10_ by 26% [157]. What is more, green spaces provide numerous profits connected with the climate (like CO_2_ sequestration and UHI mitigation), biodiversity, as well as to the human health and well-being [202]. There are also benefits, that were not known before: a positive effect of green infrastructure on the mortality and morbidity caused by COVID-19 have been observed in recent months [203].

To acquire the most from plants’ potential for PM capture, it is crucial to integrate various types of green infrastructure [204], what brings about distinct increase in LAI and capture ability. Nevertheless, there is still room for further progress in employing plants in PM reduction. The most promising expectations are related to species selection, inoculation with endophytic bacteria, and transgenic plants or microorganisms. However, the last solution is regarded controversial and does not always meet social approval, but it may be used in the vicinity of source of pollution, while maintaining spatial isolation. In order to be able to select the appropriate species for PM reduction, their PM abatement potential under local climatic conditions should be taken into consideration as well as their tolerance to stresses and VOC emissions. Inoculation of endophytic bacteria is well-documented in terms of rhizofiltration [86]. Research with the utilization of such bacteria in neutralization of air-originating contaminants is scarce, but proved to be effective [83, 205].

## Data Availability

Not applicable.

## Abbreviations

AVOCs: Anthropogenic volatile organic compounds
BVOCs: Biogenic volatile organic compounds
GHG: Greenhouse gases
GR: Green roofs
LAI: Leaf area index
MAH: Monocyclic aromatic hydrocarbons
NPP: Net primary productivity
PAH: Polycyclic aromatic hydrocarbons
PCBs: Polychlorinated biphenyls
POP: Persistent organic pollutants
PSVC: Physiognomic-structural vegetation categories
SOA: Secondary organic aerosols
SPRE: Single-pass removal efficiency
UHI: Urban heat island
VOCs: Volatile organic compounds
